# Case–control study of pathogens involved in piglet diarrhea

**DOI:** 10.1186/s13104-015-1751-2

**Published:** 2016-01-11

**Authors:** Vera L. A. Ruiz, Josete G. Bersano, Aline F. Carvalho, Márcia H. B. Catroxo, Daniela P. Chiebao, Fábio Gregori, Simone Miyashiro, Alessandra F. C. Nassar, Trícia M. F. S. Oliveira, Renato A. Ogata, Eliana P. Scarcelli, Paloma O. Tonietti

**Affiliations:** Faculty of Animal Science and Food Engineering, University of São Paulo, São Paulo, Brazil; Biological Institute, São Paulo Agency for Agribusiness Technology, Secretary of Agriculture and Food Supply, São Paulo, Brazil; Bacteriology Laboratory, Adolfo Lutz Institute, São Paulo, Brazil; São Paulo Agency for Agribusiness Technology, Secretary of Agriculture and Food Supply, São Paulo, Brazil; School of Veterinary Medicine and Animal Science, University of São Paulo, São Paulo, Brazil

**Keywords:** Case–control study, Diarrhea, Piglets

## Abstract

**Background:**

Diarrhea in piglets directly affects commercial swine production. The disease results from the interaction of pathogens with the host immune system and is also affected by management procedures. Several pathogenic agents such as *Campylobacter* spp., *Clostridium perfringens*, *Escherichia coli*, *Salmonella* spp., group A rotavirus (RV-A), coronaviruses (transmissible gastroenteritis virus; porcine epidemic diarrhea virus), as well as nematode and protozoan parasites, can be associated with disease cases.

**Results:**

All bacterial, viral, protozoan, and parasitic agents here investigated, with the exception of *Salmonella* spp. as well as both coronaviruses, were detected in varying proportions in piglet fecal samples, and positive animals were equally distributed between case and control groups. A statistically significant difference between case and control groups was found only for *Cystoisospora suis* (p = 0.034) and *Eimeria* spp. (p = 0.047). When co-infections were evaluated, a statistically significant difference was found only for *C. perfringens* β2 and *C. suis* (p = 0.014).

**Conclusions:**

The presence of pathogens in piglets alone does not determine the occurrence of diarrhea episodes. Thus, the indiscriminate use of antibiotic and anthelminthic medication should be re-evaluated. This study also reinforces the importance of laboratory diagnosis and correct interpretation of results as well as the relevance of control and prophylactic measures.

## Background

Diarrhea in piglets represents one of the major health problems affecting swine production farms. In fact, enteric infections have become one of the main causes of morbidity and mortality in neonatal farm pigs, resulting in economic losses especially when suckling and weaned piglets are affected. The disease has a multifactorial etiology influenced by environmental, management and physiological factors that include interaction of pathogens, farm procedures, and host immunity [1].

Diarrhea in piglets can be caused by several pathogenic agents, including *Campylobacter* spp., *Clostridium perfringens*, *Escherichia coli*, *Salmonella* spp., group A rotavirus (RV-A), coronaviruses (transmissible gastroenteritis virus—TGEV; porcine epidemic diarrhea virus—PEDV), as well as by nematode and protozoan parasites. However, most studies have focused on a few or only one agent and consequently our understanding of the relative importance of pathogens and other factors may have strong biases [2].

The present case–control study was carried out with piglets under field conditions in the state of São Paulo, Brazil, in order to evaluate the relative significance of pathogens in the development of intestinal disorders. It integrates microbiologic and epidemiologic data through the investigation of pathogenic agents and virulence factors in case and control animals.

## Methods

### Study design, case definition and sampling

This field-based case–control study was conducted in the state of São Paulo, in the southeastern region of Brazil, between September 2010 and July 2012. The sampling unit was a swine pen, which was defined as a group of piglets born from the same sow. Piglets with clinical signs of diarrhea represented cases, whereas piglets without clinical manifestations represented controls. The two groups were from the same farm and of similar age, but were not from the same pen.

To detect an odds ratio of 3.5 for control group exposures of 25 % or greater, with a confidence level of 95 % and a power of 80 %, the required sample size was 42 cases.

Individual fecal samples from 184 piglets (1 day to 4 weeks old) were collected from 88 pens at 16 farrow-to-finish pig farms. Among these pens, 43 represented case groups and 45 were controls.

This research was approved by an animal ethics committee subordinated to the National Council for Animal Experimentation of Brazilian Ministry of Science, Technology and Innovation (CETEA-IB 93/10).

### Laboratory methods

As summarized in Table 1, bacterial isolation, characterization of virulence and pathogenicity factors, RNA detection of viruses by RT-PCR, and coproparasitologic exams for the detection of nematode eggs as well as of protozoan cysts and oocysts were performed on the samples. Discrimination between *Cystoisospora* spp. and *Eimeria* spp. was achieved by the modified sugar flotation technique (Sheather’s sugar solution) performed after the feces with 2.5 % potassium dichromate were incubated for 5–12 days at 37 °C in a biological oxygen demand (BOD) incubator [3].

**Table 1:** Diagnostic tests performed on piglet fecal samples

Agent	Test	References
Bacteria		
*Campylobacter* spp.	Isolation	[4]
*C. coli*	Biochemical methods	[4]
*C. perfringens*	Isolation	[5]
*C. perfringens*	PCR α, β, ε e ι and cpb2 genes	[6, 7]
*E. coli*	Isolation	[8]
*E. coli*	PCR StaP, Stb e LT genes	[9]
*Salmonella* spp.	Isolation	[8]
Parasite		
Nematodes	Flotation test	[10]
Protozoa	Flotation test	[3, 11]
Virus		
Coronaviruses (PEDv and TGEv)	RT-PCR	[12]
Group A rotavirus	RT-nested-PCR	[13]

### Statistical analysis

The distribution of positive samples between cases and controls was statistically evaluated through Pearson’s Chi square or Fisher’s exact test, using the Minitab^®^ 16.1.0 software.

## Results

Bacterial, viral, protozoan, and parasitic agents that cause diarrhea, except for *Salmonella* spp. and both coronaviruses, were detected in varying proportions in the 184 examined animals (Table 2).

**Table 2:** Distribution of bacterial, viral, and parasitic agents of diarrhea in individual fecal samples from case and control piglets

Agent	Case	Control	Positive samples (%)	Positive farms
Bacteria				
*C. coli*	43	28	71 (38.59)	13
*C. perfringens* type A	26	16	42 (22.83)	10
*C. perfringens* β2	24	15	39 (21.19)	10
*E. coli* Sta toxin	5	1	6 (3.26)	4
*E. coli* Stb toxin	9	7	16 (8.69)	7
*E. coli* LT toxin	0	0	0 (0)	0
*Salmonella* spp.	0	0	0 (0)	0
Parasites				
*C. suis*	48	16	64 (34.78)	14
*Eimeria* spp.	13	2	15 (8.15)	6
Gastrointestinal Strongyles	4	3	7 (3.80)	2
Virus				
Coronavirus	0	0	0 (0)	0
Group A rotavirus	51	21	72 (39.13)	12
Total samples	123	61	184	16

When individuals were clustered into 88 groups (case, n = 43; control, n = 45), statistically significant differences were found only for protozoans (Table 3). Animals positive for bacterial and viral agents were equally distributed between case and control groups (p > 0.05) as shown in Table 3.

**Table 3:** Distribution of diarrheal agents between case and control groups

Agent	Case group (n=43)	Control group (n=45)	Pearson Chi-square	p value	Fisher’s exact test (p)
*Bacteria*					
*C. coli*	23	23	0.050	0.823	–
*C. perfringens* type A	19	15	1.092	0.296	–
*C. perfringens* β2	17	14	0.684	0.408	–
*E. coli* Sta toxin	4	1	2.057	–	0.197
*E. coli* Stb toxin	6	7	0.045	0.832	–
*E. coli* LT toxin	0	0	–	–	–
*Salmonella* spp.	0	0	–	–	–
Parasite					
*C. suis*	23ª	14ª	4.519	*0.034ª*	–
*Eimeria* spp.	8ª	2ª	4.377	–	*0.047ª*
Gastrointestinal Strongyles	2	1	0.394	–	0.612
Virus					
Coronaviruses	0	0	–	–	–
Group A rotavirus	16	15	0.145	0.704	–

^a^Statistical significant difference between case and control groups. Italic values indicate *p* < 0.05

Co-infections were analyzed, and a statistically significant difference between groups was found only for *C. perfringens* β2 and *C. suis* co-infection (p = 0.014).

## Discussion

Except for *Salmonella* spp. and both coronaviruses, all other agents commonly associated with diarrhea in pigs were detected in varying proportions in the 184 animals examined in the present study. One to six different agents were found at each farm, and one to four pathogens were detected in stool samples of infected animals.

Most studies of diarrhea in pigs have focused on a single agent, which can result in a biased view of the relevance to the disease of a particular pathogen. Calderaro et al. [14], however, studied 21 swine herds in the state of São Paulo, Brazil, from 1996 to 1997 and determined the frequency of bacterial, viral, and protozoan agents in the feces of piglets with clinical signs of diarrhea. Among the 174 samples tested in their study, 40.2 % were positive for *E. coli*, 31.6 % for *C. suis*, 10.9 % for rotavirus, and 1.2 % for *Cryptosporidium parvum*, with some samples having more than one pathogen present. Interestingly, 32.8 % of the samples tested negative for any agent. More recently, a matched case–control study evaluated the frequency of rotavirus, haemolytic *E. coli*, *C. difficile*, *C. perfringens* types A and C, *Eimeria* spp., *Cystoisospora* spp., and *Cryptosporidium* spp. associated with neonatal mild diarrhea in piglets. The study was carried out in litters of 1- to 7-day-old piglets from 28 pig farms in the state of Rio Grande do Sul, Brazil. Despite a wide range of frequencies of the different agents in case and control groups, no agent was significantly associated with diarrhea in case litters when compared to controls. Thus, the authors stressed the need for caution when interpreting laboratory diagnosis of mild diarrhea, as the detection of a single agent does not necessarily indicate that it causes the problem [15].

Fecal samples from suckling (n = 205) and weaned piglets (n = 82) with diarrhea from 24 farms in Southern Germany were examined. *C. suis* was diagnosed in 26.9 % and *C. parvum* in 1.4 % of the piglets investigated. It was found that 17.6 % of the animals were infected with enterotoxigenic *E. coli* and 4 % were positive for rotavirus. The occurrence of the pathogens was significantly associated with the age of the animals examined [16].

Rotaviruses represent one of the most frequently detected viral agents associated with diarrhea in swine worldwide, especially in 1- to 4-week-old pigs [17, 18]. In 75 % of the visited farms, almost 40 % of stool samples tested were positive for RV-A, indicating the high frequency of this viral infection among piglets in Brazil. Nevertheless, this viral agent was equally distributed between case and control groups. According to Svensmark et al. [19], rotaviruses are more frequently detected in semiliquid and loose stools than in normal or watery stools. However, when rotavirus infection was studied in 1090 litters from 26 intensively managed Danish sow herds, an association between virus detection and diarrhea could not be demonstrated [19]. On the other hand, a significant difference has been reported regarding the frequency of RV-A in diarrheic and non-diarrheic fecal samples [20]. These previous results, together with ours, indicate that in spite of the wide distribution of rotaviruses, additional factors may be involved in the development of clinical cases.

Negative RT-PCR results obtained in this study for coronaviruses confirm previous reports of the absence of serological evidence of these infections in Brazilian pig herds [14, 21, 22].

Although campylobacteriosis is one of the most common causes of diarrhea in humans, the role of *Campylobacter* spp. in swine gastrointestinal disorders is still controversial. In 2005, a study suggested that pigs represent an important *C. coli* reservoir in Germany. However, the clinical relevance of this finding was not evaluated, because this broad study aimed at monitoring foodborne pathogens [23]. An experimental infection conducted to evaluate the colonization and translocation ability of a porcine strain of *C. coli* showed that all ten infected animals remained in very good health, although overall fecal consistency, rated on a five-point scale, decreased from 4.0 to 3.5 over 4 days [24]. In another study, no statistically significant difference was found in the number of pigs with *Campylobacter* spp. between diarrheic and healthy animals. However, CFU counts were significantly different in the two groups, suggesting that *Campylobacter* spp. may play a role as a cofactor in pig diarrhea [25]. Despite the fact that *C. coli* was the most frequent bacteria found in the present study, with almost 40 % of samples positive and 81.25 % of farms positive, no difference in frequency was found between case and control groups, which is in agreement with previous reports. Nevertheless, in one industrialized well-managed indoor farm, we found that all animals that were positive only for *C. coli* had severe diarrhea, while control animals were negative for all pathogens tested. Altogether, these results suggest that *C. coli* may play a role in pathogenesis, although it is important to consider other agents or factors not tested in this work.

*Clostridium perfringens* type A was found in almost 23 % of diarrheic and non-diarrheic samples from 62.5 % of the farms, yet again there was no statistical difference between case and control groups, even when the subgroup of *C. perfringens* carrying the cpb2 gene was investigated (21.2 % positive samples). Chan et al. [26] identified *C. perfringens* as the causal agent of gastrointestinal tract illness in 28 of 237 studied cases, and genotyping of 17 strains showed that they belonged to toxinotype A and had the cpb2 gene. In another study, intestinal positivity for *C. perfringens* was detected in 73 % of diarrheic and 78 % of healthy piglets. Those bacteria were mostly present in the intestinal lumen. In 20 % of diarrheic and 30 % of healthy animals, bacteria were found within the mucus layer and in direct contact with the intestinal epithelium. However, presence and location of *C. perfringens* in the intestinal tissue did not significantly correlate with histological lesions [27]. Other authors necropsied and took intestinal samples from 46 piglets from 10 farms with a consistent history of type-A *C. perfringens* neonatal diarrhea. Samples were compared to those from an unaffected cohort of piglets. Based on the number of intestinal bacteria, presence of consensus cpb2 in *C. perfringens* isolates, expression of cpb2 in piglet intestines, and known or unknown causes of diarrhea, these investigators were unable to distinguish between healthy and diarrheic piglets [28]. The role of cpb2-harboring *C. perfringens* in the development of diarrhea was also investigated through the assessment of cytotoxicity to porcine IPI-21 and human Caco-2 cell-lines. Supernatants of cpb2-harboring *C. perfringens* were cytotoxic to both cells to variable extents. However, toxin removal by anti-beta 2 toxin antibodies or degradation by trypsin did not reduce the cytotoxic effect of supernatants [29]. These results indicate the need for further studies focused on elucidating the role of cpb2-positive *C. perfringens* type A in neonatal diarrhea.

Neonatal intestinal infection with *E. coli* causes severe diarrhea and frequently kills piglets [30]. Different strains are described as responsible for clinical conditions, especially strains that produce enterotoxins such as the heat-labile enterotoxin (LT) and the heat-stable enterotoxin (ST) [31, 32]. In the present study, less than 12 % of examined samples were positive for ST (STa or STb), and no statistically significant difference between case and control groups was found. In Canada, from 2001 to 2010, 31 % of 237 samples submitted for gastrointestinal disease laboratory diagnoses had enterotoxigenic *E. coli* (ETEC) infection, and ETEC was less likely to be recovered when *C. difficile*, *C. perfringens* or rotavirus were detected (p < 0.05) [26]. In four commercial Danish swine herds, intestinal positivity for *E. coli* was found in 88 % and 80 % of the small intestines of diarrheic and non-diarrheic piglets, respectively. Nevertheless, diarrheic piglets had large numbers of *E. coli* more frequently than non-diarrheic piglets [27]. Our results showed that 25 and 43.75 % of the farms were positive for *E. coli* STa and STb toxins, respectively, which represents a risk of outbreaks and of selection of resistant pathogenic strains.

*Salmonella* spp. was not found in the examined samples. These findings were expected, because this agent is not usually found in such young piglets [33, 34].

The equal distribution of bacterial agents between groups may have resulted from the extensive use of antibiotics in Brazilian swine production. This finding reinforces the need for a reassessment of the use of antibiotics in food-producing livestock.

Based on parasitological analysis, only two farms and 3.8 % of samples were positive for nematode eggs, and no statistically significant difference between case and control groups was found. These results suggest that systematic use of anthelminthic drugs associated with indoor housing systems and hygiene procedures can control infection by breaking the chain of transmission. However, animal welfare concerns are leading to changes in management practices. During recent decades, the number of organic and “green” swine herds has increased, and this may be an indication that former risk factors could arise again [35].

The detection frequency of *Eimeria* spp. was 8.15 % among tested samples (37.5 % of farms), and there was a statistically significant difference between case (18.60 %) and control (4.44 %) groups (p = 0.047). Some authors consider *Eimeria* spp. infection in piglets an uncommon cause of clinical signs [36–39]. However, more recently, *Eimeria* spp. was identified in 13 % of fecal samples from suckling piglets with diarrhea [40].

*C. suis* was the most commonly detected coccidian agent, present in 34.78 % of samples and widespread in the studied farms (87.5 %). A significant difference was again observed between case (53.49 %) and control (31.11 %) groups (p = 0.034). The ability of *C. suis* to cause diarrhea in piglets is well documented [41, 42], as is its frequency of infection in young piglets: 17.3 % in the Republic of Korea [43], 53.8 % in Germany [44], 31.6 % in Brazil [14], 6.3 % in Canada [26], and 8.9 % in Cuba [45]. Our results differ from those of another study that was recently published in Brazil in which no statistical difference between case and control groups was found [15]. Methodological aspects of the two studies could explain the differing results. We collected samples from 1-day- to 4-week-old animals, while piglets between 1 and 7 days of age were sampled in the previous Brazilian study. Age of piglets seems to be crucial for the outcome [46]. *C. suis* infections in piglets undoubtedly have a high impact. However, encouraging the use of drugs to control this agent could lead to abuses similar to those seen with antibiotic use. Coccidian oocysts are generally regarded as relatively resistant to environmental factors and apt to survive for considerable periods. However, high temperature (25–30 °C) in combination with low relative humidity (53–62 %) rapidly reduces the viability of *C. suis* oocysts. This finding might point to a possible control mechanism requiring only some environmental control and proper management of farrowing pens, like by allowing a few extra days in-between litters or by increasing desiccation somehow, might be able to reduce the number of infective *C. suis* oocysts that has escaped pen cleaning [47].

According to Mengel et al. [48], newborn piglets exposed to natural *C. perfringens* type A infection and to low-level experimental infection with *C. suis* showed an increase in clinical disease, mortality, and metabolically active *C. perfringens* type A. In the present study, analyses of 28 possibilities of co-infection by two agents and 55 possibilities of co-infection by three agents identified a potential for worsening conditions only in the combination of *C. suis* and *C. perfringens* type A (cpb2 gene) (p = 0.014), corroborating the hypothesis that simultaneous infection with these agents soon after birth may lead to an increase in the severity of clinical disease in piglets [48].

Recently, a non-hemorrhagic diarrhea during the first week of life, with no detection of known infectious agents and characterized by a milk-filled stomach and flaccid intestines at necropsy was described. The syndrome is not related to starvation or infection by enterotoxigenic *E. coli*, *C. perfringens* type A or C, *C. difficile*, rotavirus, coronavirus, *Cryptosporidium* spp., *Giardia* spp., *C. suis* or *Strongyloides ransomi*. The existence of neonatal diarrhea with unspecific lesions and without known pathogens is not a new phenomenon [49], but this study also reinforces the importance of laboratory diagnosis and correct interpretation of results as well as the relevance of control and prophylactic measures.

## Conclusions

The presence of known pathogens in piglets alone does not seem to determine the occurrence of diarrhea. The indiscriminate use of antibiotic and anthelminthic medication should be reassessed. The importance of laboratory diagnosis and correct interpretation of data as well as the relevance of control and prophylactic measures should be reinforced.

The aim of this case–control study was to assess the association between a variety of pathogens and the occurrence of diarrhea episodes in 1-day- to 4-week-old piglets. Statistically significant differences in pathogen frequency between animals in case and control groups were found for the protozoan agents *C. suis* and *Eimeria* spp., and *C. suis* and *C. perfringens* type A co-infection. This finding may indicate that coccidian agents should be independently considered in disease control and monitoring programs.

## References

[1] Zlotowski P, Driemeier D, Barcellos DESN (2008). Patogenia das diarreias dos suínos: modelos e exemplos. Acta Sci Vet.

[2] Zimmerman JJ, Karriker LA, Ramirez A, Schwartz KJ, Stevenson GW (2012). Diseases of swine.

[3] Vetterling JM (1965). Coccidia (Protozoa: Eimeriidae) of swine. J Parasitol.

[4] OIE. World Organisation for Animal Health. Chapter 2.9.3.—*Campylobacter jejuni* and *Campylobacter coli*. OIE Terrestrial Manual 2008. p. 1185–91. Disponível em: http://www.oie.int/fileadmin/Home/eng/Health_standards/tahm/2.09.03_CAMPYLO.pdf. Acesso em: fev. 2010.

[5] Baldassi L, Barbosa ML, Bach EE, Iaria ST (2002). Toxigenicity characterization of *Clostridium perfringens* from bovine isolates. J Venom Anim Toxins.

[6] Meer RR, Songer JG (1997). Multiplex PCR method for genotyping *Clostridium perfringens*. Am J Vet Res.

[7] Bueschel DM, Jost BH, Billington SJ, Trinh HT, Songer G (2003). Prevalence of cpb2, encoding beta2 toxin, in *Clostridium perfringens* field isolates: correlation of genotype with phenotype. Vet Microbiol.

[8] Winn WC, Koneman EW (2006). Koneman’s color atlas and textbook of diagnostic microbiology.

[9] Macêdo NR, Menezes CPL, Lage AP, Ristow LE, Reis A, Guedes RMC (2007). Detecção de cepas patogênicas pela PCR multiplex e avaliação da sensibilidade a antimicrobianos de *Escherichia coli* isoladas de leitões diarréicos. Arq Bras Med Vet Zootec.

[10] Gordon HML, Whitlock AV (1939). A new technique for counting nematode eggs in sheep feces. J Counc Sci Ind Res..

[11] Benbrook EA, Sloss MW (1955). Veterinary clinical parasitology.

[12] Brandão PE, Gregori F, Villarreal LYB, Rosales CAR, Soares RM, Jerez JA (2005). A nested polymerase chain reaction to bovine coronavirus diagnosis based on the RNA-dependent RNA-polymerase gene. Virus Rev Res.

[13] Salem ANB, Chupin SA, Bjadovskaya OP, Andreeva OG, Mahjoub A, Prokhvatilova LB (2010). Multiplex nested RT-PCR for the detection of porcine enteric viruses. J Virol Methods.

[14] Calderaro FF, Baccaro MR, Moreno AM, Ferreira AJP, Jerez AJ, Pena HJF (2001). Frequência de agentes causadores de enterites em leitões lactentes provenientes de sistemas de produção de suínos do Estado de São Paulo. Arq Inst Biol.

[15] Lippke RT, Borowski SM, Marques SMT, Paesi SO, Almeida LL, Moreno AM, Corbellini LG, Barcellos DESN (2011). Matched case-control study evaluating the frequency of the main agents associated with neonatal diarrhea in piglets. Pesq Vet Bras.

[16] Wieler LH, Ilieff A, Herbst W, Bauer C, Vieler E, Bauerfeind R, Failing K, Klös H, Wengert D, Baljer G, Zahner H (2001). Prevalence of enteropathogens in suckling and weaned piglets with diarrhoea in Southern Germany. J Vet Med B.

[17] de San Sigolo, Juan C, Bellinzoni RC, Mattion N, La Torre J, Scodeller EA (1986). Incidence of group A and atypical rotaviruses in Brazilian pig herds. Res Vet Sci.

[18] Estes MK, Kapikian AZ, Fields BN, Knipe DM, Howley PM, Griffin DE, Lamb RA, Martin MA, Roizman B, Straus SE (2007). Rotaviruses and their replication. Fields virology.

[19] Svensmark B, Nielsen K, Dalsgaard K, Willeberg P (1989). Epidemiological studies of piglet diarrhea in intensively managed Danish sow herds. III. Rotavirus infection. Acta Vet Scand.

[20] Linares RC, Barry AF, Alfieri AF, Médici KC, Feronato C, Grieder W, Alfieri AA (2009). Frequency of group a rotavirus in piglet stool samples from non-vaccinated Brazilian pig herds. Braz Arch Biol Technol.

[21] Brentano L, Ciacci-Zanella JR, Mores N, Piffer IA. Levantamento Soroepidemiológico para Coronavírus Respiratório e da Gastroenterite Transmissível e dos Vírus de Influenza H3N2 e H1N1 em Rebanhos Suínos no Brasil. Comunicado Técnico EMBRAPA, 2002. n. 306.

[22] Barthasson DL, Brito WMED, Sobestiansky J, Caixeta SPMB, Miranda TMT, Silva LA (2009). Ocorrência de infecção por parvovírus suíno e gastrenterite transmissível em suínos, criados de forma extensiva, em Goiás. Arq Bras Med Vet Zootec.

[23] Alter T, Gaull F, Kasimir S, Gürtler M, Mielke H, Linnebur M, Fehlhaber K (2005). Prevalences and transmission routes of *Campylobacter* spp. strains within multiple pig farms. Vet Microbiol.

[24] Bratz K, Bücher R, Gölz G, Zakrzewski SS, Janczyk P, Nöckler K, Alter T (2013). Experimental infection of weaned piglets with *Campylobacter coli*—excretion and translocation in a pig colonisation trial. Vet Microbiol.

[25] Modolo JR, Margato LFF, Gottscharlk AF, Lopes CAM (1999). Incidence of *Campylobacter* in pigs with and without diarrhea. Rev Microbiol.

[26] Chan G, Farzan A, DeLay J, McEwen B, Prescott JF, Friendship RM (2013). A retrospective study on the etiological diagnoses of diarrhea in neonatal piglets in Ontario, Canada, between 2001 and 2010. Can J Vet Res.

[27] Jonach B, Boye M, Stockmarr A, Jensen TK (2014). Fluorescence in situ hybridization investigation of potentially pathogenic bacteria involved in neonatal porcine diarrhea. BMC Vet Res.

[28] Farzan A, Kircanski J, DeLay J, Soltes G, Songer JG, Friendship RM, Prescott JF (2013). An investigation into the association between cpb2-encoding *Clostridium perfringens* type A and diarrhea in neonatal piglets. Can J Vet Res.

[29] Allaart JG, van Asten AJAM, Vernooij JCM, Gröne A (2014). Beta2 toxin is not involved in in vitro cell cytotoxicity caused by human and porcine cpb2-harbouring *Clostridium perfringens*. Vet Microbiol.

[30] Moon HW, Schineider RA, Mosely SL (1986). Comparative prevalence of four enterotoxin genes among *Escherichia coli* isolates from swine. Am J Res.

[31] Dorner F (1975). *Escherichia coli* enterotoxin. Purification and partial characterization. J Biol Chem.

[32] Clements JD, Finkelstein RA (1979). Isolation and characterization of homogeneous heat-labile enterotoxins with high specific activity from *Escherichia coli* cultures. Infect Immun.

[33] Songer JG, Post KW. Veterinary microbiology. Bacterial and fungal agents of animal disease. Ed. St Louis: Elsevier Saunders; 2005. https://elsevier.ca/product.jsp?isbn=9780721687179

[34] Schwarz P, Hirose F, Kolb J, Calveyra J, Barcellos DESN, Cardoso M. Longitudinal study of *Salmonella enterica* infection in a swine herd in Southern Brazil. In: IPVS congress, 2006, Copenhagen. Proceedings. Iowa: IPVS, 2006.

[35] Nansen P, Roepstorff A (1999). Parasitic helminths of the pig: factors influencing transmission and infection levels. Int J Parasitol.

[36] Lindsay DS, Blagburn BL, Boosinger TR (1987). Experimental *Eimeria debliecki* infections in nursing and weaned pigs. Vet Parasitol.

[37] Koudela B, Vitovec J (1992). Biology and pathogenicity of *Eimeria spinosa* Henry, 1931, in experimentally infected pigs. Int J Parasitol.

[38] Daugschies A, Imaron S, Ganter M, Bollwahn W (2004). Prevalence of *Eimeria* app. in sows at piglet-producing farms in Germany. J Vet Med B.

[39] Karamon J, Ziombo I, Cencek T (2007). Prevalence of *Isospora suis* and *Eimeria* spp. in suckling piglets and sows in Poland. Vet Parasitol.

[40] Zhang WJ, Xu LH, Liu YY, Xiong BQ, Zhang QL, Li FC, Song QQ, Khan MK, Zhou YQ, Hu M, Zhao J (2012). Prevalence of coccidian infection in suckling piglets in China. Vet Parasitol.

[41] Stuart BP, Lindsay DS, Ernst JV, Gosser HS (1980). *Isospora suis* enteritis in piglets. Vet Pathol.

[42] Harleman JH, Meyer RC (1983). *Isospora suis* in piglets. A review. Vet Quart.

[43] Chae C, Kwon D, Kim O, Min K, Cheon DS, Choi C, Kim B, Suh J (1998). Diarrhea in nursing piglets associated with coccidiosis: prevalence, microscopic lesions and coexisting microorganisms. Vet Rec.

[44] Meyer C, Joachim A, Daugschies A (1999). Occurrence of *Isospora suis* in larger piglet production units and on specialized piglet rearing farms. Vet Parasitol.

[45] Rodríguez PYF, Martin LOM, Muñoz EC, Imberechts H, Butaye P, Goddeeris BM, Cox E (2013). Several enteropathogens are circulating in suckling and newly weaned piglets suffering from diarrhea in the province of Villa Clara, Cuba. Trop Anim Health Prod.

[46] Mundt HC, Joachim A, Daugschies A, Zimmermann M (2003). Population biology studies on *Isospora suis* in piglets. Parasitol Res.

[47] Langkjær M, Roepstorff A (2008). Survival of *Isospora suis* oocysts under controlled environmental conditions. Vet Parasitol.

[48] Mengel H, Kruger M, Kruger MU, Westphal B, Swidsinski A, Schwarz S, Mundt HC, Dittmar K, Daugschies A (2012). Necrotic enteritis due to simultaneous infection with *Isospora suis* and clostridia in newborn piglets and its prevention by early treatment with toltrazuril. Parasitol Res.

[49] Kongsted H, Jonach B, Haugegaard S, Angen O, Jorsal SE, Kokotovic B, Larsen LE, Jensen TK, Nielsen JP (2013). Microbiological, pathological and histological findings in four Danish pig herds affected by a new neonatal diarrhoea syndrome. BMC Vet Res.

